# Association Between Body Mass Index and Caries Frequency Among Zahedan Elementary School Children

**DOI:** 10.5812/ijhrba.10220

**Published:** 2013-12-22

**Authors:** Touran Shahraki, Mansour Shahraki, Salehe Omrani Mehr

**Affiliations:** 1 Department of Pediatrics, Faculty of Medicine, Zahedan University of Medical Sciences, Zahedan, IR Iran; 2 Children and Adolescent Health Research Center, Zahedan University of Medical Sciences, Zahedan, IR Iran; 3 Department of Nutrition, Faculty of Medicine, Zahedan University of Medical Sciences, Zahedan, IR Iran; 4 Zahedan University of Medical Sciences, Zahedan, IR Iran

**Keywords:** Body Mass Index, Dental Caries, child

## Abstract

**Background:**

A balanced nutrition schedule provides the essential substances for proper oral health.

**Objectives:**

The aim of this study was to investigate the association between dental caries and body mass index in 6-11 year-old children in Zahedan.

**Materials and Methods:**

In this cross-sectional study 1213 children (670 girls, 543 boys) were included. Body mass index (BMI) and clinical examination for determination of DFT (decay filling teeth) index (based on WHO criteria) were taken. Collected data were analyzed using the t-test, chi-square and ANOVA.

**Results:**

Among children, 20.8% had low weight, 66.3% normal weight, 7.8% were overweight and 5.1% obese. In the low weight, normal weight, overweight, and obese groups, the mean ± SD values for DFT were: 0.63 ± 1.1, 0.88 ± 1.36, 1.16 ± 1.33, and 0.87 ± 1.31, respectively. There was a significant association between BMI and DFT (P = 0.005). The overweight group had higher DFT compared to the low and normal weight groups. 13.5% of low weight, 12.2% of normal weight, 14.7% of overweight and 22.6% of obese children had DFT = 0. There was no statistical association between BMI and being caries free (P = 0.4).

**Conclusions:**

The mean DFT in the overweight group was higher than low and normal weight groups. There was a statistically significant association between BMI and DFT.

## 1. Background

Dental caries is one of the major health problems in the world (1). Its impact on individuals and communities as well as functional impairments and reduced quality of life are considerable. In many developing countries, treatment of dental caries in children would exceed the total child health care budget (2). Although different national preventive programs have significantly decreased the dental decay, caries still remain a major national public health problem. Data from dental control and prevention centers shows that prevalence of untreated caries in primary dentition among 6-11 year-old US children is 25%. Almost 60% of 12 to 15-year-old children have had at least one carious tooth or a restoration and this increases to 78% by the age of 16 (3). Data from surveys in Iran represent a marked decline in dental caries (4).

An association between weight and oral health in children has had controversial results. On one hand, consumption of soft drinks and fast food as well as lack of activity and exercise contributed to the increasing number of overweight individuals around the world (5). Overweight children have been associated with prolonged exposure to carbohydrates. Intake of refined carbohydrates especially sugars are well documented in the literature (6). In a study by Willerhausen et al. 1290 elementary school children (648 boys, 642 girls) were examined. The study showed that 3.6% of the children were underweight, 74.8% had normal weight, 11.9% were overweight, and 9.7% were obese. A significant association between high weight and caries frequency was observed (7). However, one Brazilian study concluded that there was no statistically significant association between dental caries and obesity in adolescents aged 12 to 15 years (8).

## 2. Objectives

Although surveys on Iranians show improvements in oral health education and dental services, various regions of a country may have different dental caries frequencies compared to other parts due to different conditions and access to dental care. No information on the association of BMI and dental caries is available from Zahedan. The purpose of this study was to determine the relationship between BMI-for-age and dental status of primary school children in Zahedan, Iran.

## 3. Materials and Methods

This cross-sectional study was conducted on 1213 primary school children (670 females, 543 males) aged 6-11 years, in Zahedan. Using a random cluster sampling, 12 girls and boys primary schools (classes I-V for children aged 6-11 years) in the study area with different social backgrounds were screened. From a total of 1233 children at the beginning of the study, 20 children had chronic problems and were excluded.

The body weights were recorded to the nearest 100 g using a standard beam balance scale (Seca). Subjects had light dresses. The scale was calibrated at the beginning of each working day and at frequent intervals throughout the day. The body heights were recorded to the nearest 0.5 cm without shoes.

BMI-for-age [(weight in kilograms)/(height in meters) ²] was calculated. Underweight was defined as BMI-for-age < 5th percentile, “normal” was defined as 5th percentile < BMI-for-age < 85th percentile, “at risk of overweight” was defined as 85th percentile < BMI-for-age < 95th percentile, and “Obese” was defined as BMI-for-age > 95th percentile (9). Clinical examinations were carried out by two dentists who used the WHO criteria for diagnosis of dental caries (10). The DMFT (decayed, missing and filled teeth) values were also derived. The examination was carried out using a plane mouth mirror and cotton rolls to remove any plaque or debris where necessary and recorded on special charts. No radiographs were taken. White spots were not considered as decayed in this study. Missing teeth were not marked correspondingly, since no definite statement could be made without proper anamnesis to determine whether the tooth really existed or not, or if an early extraction had taken place. To estimate the caries frequency, the DFT (decay filling teeth) index was used for the permanent dentition since it gives a good insight into the state of decay in the patient (10). Dental examinations of children were only conducted if a written consent was obtained from the parents and/or guardians. Calibration was made between two examiners. To achieve the best coordination, all dental examinations, based on the above criteria, were done by one examiner, and body weight and height were recorded by another examiner.

The study was confirmed by the research committee of the Faculty of Dentistry, Zahedan University of Medical Sciences.

### 3.1. Statistical Analysis

Means and standard deviations were calculated to express the DFT values. The t-test was applied to study the association of the dental caries prevalence with gender and age. ANOVA test was used for the relationship between DFT and BMI. P ≤ 0.05 was considered statistically significant.

## 4. Results

A total of 1213 children were present in this study (670 (55.2%) girls and 543 (44.8%) boys). Thirteen cases (1 boy and 12 girls) were 6, 223 children (86 boys and 137 girls) were 7, 271 children (127 boys and 144 girls) were 8, 206 children (105 boys and 101 girls) were 9, 238 children (102 boys and 136 girls) were 10, and 261 children (121 boys and 140 girls) were 11 years old.

Five percent of the children in the study (n = 62; 32 boys and 30 girls) were obese, 7.8% (n = 95; 33 boys and 62 girls) were overweight, 66% (n = 804; 348 boys and 456 girls) had normal weight, and 20% (n = 252; 130 boys and 122 girls) were underweight. There was a statistically significant association between BMI-for-age and gender (P = 0.01).

**Table 1.** Distribution of BMI (Body Mass Index) and Gender in the Study Subjects

BMI for Age	Underweight	Normal	Overweight	Obese	Total
**Female, No. (%)**	122 (48.4)	456 (56.7)	62 (65.3)	30 (48.4)	670
**Male, No. (%)**	130 (51.6)	348 (43.3)	33 (34.7)	32 (51.6)	543
**Total, No. (%)**	252 (100)	804 (100)	95 (100)	62 (100)	1213

There were more obese and overweight girls than boys in the study (P = 0.01). The gender differences of the carious lesions frequencies in relation with the BMI values resulted in a slightly higher occurrence of carious lesions in girls. The boys and girls had a mean of 0.7 ± 1.24 and 0.9 ± 1.36 for carious lesions (DFT values) in their permanent dentition, respectively (P = 0.05).

Distribution of the children according to the DFT index and BMI is given in Table 2. Considering the BMI-for-age values, 98 of the children had normal weight and among underweight cases, 34 were caries free; whereas only 28 subjects in the overweight and obese groups were caries free. There was a significant association between BMI and DFT (P = 0.005).

**Table 2.** Mean and Standard Deviation of DFT/DFT Indices and Number (%) of 6-11 Year-Old Caries Free Children According to the BMI-for-Age values

BMI for Age	Mean ± SD	DFT = 0, No. (%)
**Underweight**	3.82 ± 2.94	34 (13.5)
**Normal**	3.42 ± 2.70	98 (12.2)
**Overweight**	2.06 ± 2.12	14 (14.7)
**Obese**	2.16 ± 2.66	14 (22.6)
**Total**	3.3 ± 2.75	160

Daily consumption of sweet products did not have any relationship with dental caries and BMI. Watching TV was more frequent in girls than boys (P = 0.0001). DFT decreased with increased tooth brushing (P = 0.0001).

**Table 3.** The Correlation Between DMFT^a^ and the Brushing Index

Index, No./d	DMFT, Mean ± SD
**Once**	0.8 ± 1.2
**Twice**	0.7 ± 1.3
**Three times**	0.5 ± 1.09
**Sometimes**	0.9 ± 1.3
**Never in one day**	1.3 ± 1.4
**Total**	0.85

^a^ Abbreviation: DMFT; decayed, missing, filled, teeth.

## 5. Discussion

There have been no oral epidemiological studies determining the association between BMI and DFT in Zahedan. Considering this issue, the present study intended to provide such information. The studied subjects were 13% obese and overweight, 66% had normal weight, and 20% were underweight. Our results showed a significant association between BMI-for-age and gender (more obese and overweight girls than boys; P = 0.01). There was a slightly higher occurrence of carious lesions in girls. This research also revealed that only 37% children in overweight and obese groups were caries free. Caries prevalence was substantially lower in children with increased tooth brushing (P = 0.0001).

In a study by Hilgers KK et al. on 178 children aged 8 to 11 years, caries severity averages were analyzed. The results showed that the mean caries for permanent molars significantly increased with increased BMI, which is similar to our results (11). In another study on 1290 elementary school children (648 boys and 642 girls) in Germany, the number of natural healthy teeth decreased with age (P = 0.001) and BMI (P = 0.0061) and was different between girls and boys (P = 0.0334). This study demonstrated a significant association between caries frequency and weight in school children (7). Another research on Turkish school children revealed that children with low body weight were at a higher risk of developing dental caries than overweight/obese children (12).

In an Iranian survey, the percentages of caries-free children (deciduous and permanent teeth) among 6 and 9 years old were reported as 13.8 and 11.5 respectively, and more than 50% of 12-year-old children had caries experience, with the decay component being the greatest cause. Although improvement in DFT index was shown as marked decline in dental caries from DFT of 4 to 1.5 in 12-year-old children, the authors emphasized that the general level of oral health is still not satisfactory especially among children and recommended that interventions should be taken (13). In another cross-sectional study in Iran, a total of 1003 children (6-11 years old) were screened. Most of the subjects were overweight and obese (16.9% and 67.1%). There was no statistically significant association between BMI-for-age and DFT. Among the cases, 27.7%, 14% and 37.2% of children with normal weight, at risk of being overweight and overweight respectively, were caries free. There was a statistically significant association between BMI-for-age and being caries free (P = 0.0001) (14). In addition, results of a study in Hamadan showed that the highest DFT was seen in normal weight and the lowest DFT in overweight children. There was not a statistically significant relationship between high weight and caries frequency in the first and permanent dentitions (15). Our study did not demonstrate a significant association between BMI-for-age and being caries free.

The results of our study showed that the number of tooth brushing sessions in a day could affect the DFT. Brushing three times was more effective than never in one day (1.09 ± 0.5 vs. 1.4 ± 1.3 respectively). Daily tooth brushing behaviors and age of brushing initiation with parental supervision have been reported by several studies as strong caries prevention methods (16, 17). In a research on elementary school children, prevalence of caries was substantially lower in children with increased tooth brushing (7). In a prevalence study on 281 children aged 5 - 14 years, the frequency of tooth brushing and sweet consumption as well as their impacts on the prevalence of caries were studied. The children who never brushed their teeth had a 40% chance of early caries, while those who brushed their teeth several times a day had a 15.3% chance. The authors believed that the positive effect of tooth brushing was superior to that of a correct diet (18).

Finally, we analyzed the caries prevalence rates using DFT, but there were other factors such as cultural and hygienic habits (19) that we did not evaluate. More studies in this regard are recommended. Determining other factors linked to dental caries is of great importance as they are temporal and geographic stability and as a tool to tailor proper health education campaigns to combat oral health problems especially among those who are in need.

The results of this study indicate a possible association between high weight and caries. Besides numerous other risk factors, high weight can also be responsible for increased number of carious lesions. In future preventive programs, strategies should aim at nutrition control to avoid high weight as well as carries.

## References

[1] World Health Organization (2000). Global Oral Health Data Bank..

[2] Yee R, Sheiham A (2002). The burden of restorative dental treatment for children in Third World countries.. Int Dent J..

[3] Beltran-Aguilar ED, Barker LK, Canto MT, Dye BA, Gooch BF, Griffin SO (2005). Surveillance for dental caries, dental sealants, tooth retention, edentulism, and enamel fluorosis--United States, 1988-1994 and 1999-2002.. MMWR Surveill Summ..

[4] Pakshir HR (2004). Oral health in Iran.. Int Dent J..

[5] Willershausen B, Haas G, Krummenauer F, Hohenfellner K (2004). Relationship between high weight and caries frequency in German elementary school children.. Eur J Med Res..

[6] Kopycka-Kedzierawski DT, Auinger P, Billings RJ, Weitzman M (2008). Caries status and overweight in 2- to 18-year-old US children: findings from national surveys.. Community Dent Oral Epidemiol..

[7] Willerhausen B, Blettner M, Kasaj A, Hohenfellner K (2007). Association between body mass index and dental health in 1,290 children of elementary schools in a German city.. Clin Oral Investig..

[8] Moreira PV, Rosenblatt A, Severo AM (2006). Prevalence of dental caries in obese and normal-weight Brazilian adolescents attending state and private schools.. Community Dent Health..

[9] Lysen LK, Israel DA, Mahan LK, Stump S, Raymond JL (2012). Nutrition in weight management.. Krause's Food and the Nutrition Care Process..

[10] (1997). Oral health surveys. Basic Methods..

[11] Hilgers KK, Kinane DE, Scheetz JP (2006). Association between childhood obesity and smooth-surface caries in posterior teeth: a preliminary study.. Pediatr Dent..

[12] Koksal E, Tekcicek M, Yalcin SS, Tugrul B, Yalcin S, Pekcan G (2011). Association between anthropometric measurements and dental caries in Turkish school children.. Cent Eur J Public Health..

[13] Pakshir HR (2003). Dental education and dentistry system in Iran.. Med Princ Pract..

[14] Sadeghi M, Alizadeh F (2007). Association between Dental Caries and Body Mass Index-For-Age among 6-11-Year-Old Children in Isfahan in 2007.. J Dent Res Dent Clin Dent Prospects..

[15] Mojarad F, Maybodi MH (2011). Association between dental caries and body mass index among hamedan elementary school children in 2009.. J Dent (Tehran)..

[16] Petersen PE (2005). Sociobehavioural risk factors in dental caries - international perspectives.. Community Dent Oral Epidemiol..

[17] Harris R, Nicoll AD, Adair PM, Pine CM (2004). Risk factors for dental caries in young children: a systematic review of the literature.. Community Dent Health..

[18] Pita-Fernandez S, Pombo-Sanchez A, Suarez-Quintanilla J, Novio-Mallon S, Rivas-Mundina B, Pertega-Diaz S (2010). [Clinical relevance of tooth brushing in relation to dental caries].. Aten Primaria..

[19] Petersen PE (2004). Challenges to improvement of oral health in the 21st century-the approach of the WHO Global Oral Health Programme.. Int Dent J..

